# Utilizing Social Media for Information Dispersal during Local Disasters: The Communication Hub Framework for Local Emergency Management

**DOI:** 10.3390/ijerph182010784

**Published:** 2021-10-14

**Authors:** Dionne Mitcham, Morgan Taylor, Curtis Harris

**Affiliations:** Institute for Disaster Management, College of Public Health, University of Georgia, Athens, GA 30602, USA; domitcham@gmail.com (D.M.); Morgan.Taylor@uga.edu (M.T.)

**Keywords:** emergency management, social media, risk communication, crisis communication, local government

## Abstract

In today’s society, the use of social media has increased the public’s desire to receive information quickly and to be able to interact with communicators. During a disaster, the trend to turn to social media for information has risen in popularity. Society’s reliance on social media and quick access to information has led the field of emergency management and the role of a Public Information Officer to adapt to include social media as a crisis communication channel for information dispersal. Existing frameworks for the use of social media as a channel for crisis communications provide guidance for emergency management agencies across all levels of government but fail to account for the varying access to communication resources at the local level. Due to the differing access to communication resources and unique relationships with stakeholders at the local level, there is a need for guidance on how local emergency management agencies can use social media to disperse essential information. The proposed Communication Hub Framework utilizes local emergency management professionals’ relationships with key community stakeholders to aid in the distribution of essential information to community members via social media during a disaster.

## 1. Introduction

Since the introduction of Facebook in 2004 and Twitter in 2006, social media has risen in popularity as a source of news, information, and entertainment in everyday life [1,2,3]. The reliance on social media in today's society combined with the hallmark introduction of the utilization of social media as a communication tool in the response to the Boston Marathon bombings in 2013 has led to the increasingly popular trend of disseminating and correcting disaster-related information via social media [4]. Previous instances of miscommunication and the spreading of rumors via social media during disasters, such as Hurricane Harvey and the Boston Marathon bombing, has demonstrated the increasing need for guidance and frameworks directing the use of social media as a communication tool at all levels of the field of Emergency Management (EM). Although federal organizations have attempted to meet the need by releasing guidelines on the use of social media, such as the Crisis and Emergency Risk Communications (CERC) framework and the Social Media Emergency Management (SMEM) Guidance Tool, the frameworks were crafted to be used at all levels of EM and do not offer tailored recommendations for social media use at the local level. Local EM offices often have varying access to certain communication resources that state and federal emergency management organizations have, such as risk communicators, social media strategists, and full-time Public Information Officers (PIOs) [5]. Additionally, local EM offices and agencies have unique relationships with community partnerships and stakeholders compared to state and federal agencies [6]. The current available frameworks and social media guidelines fail to account for local EM agencies’ differing access to communication resources and do not utilize the close relationships local EM agencies have with local stakeholders.

Given the current lack of guidance specifically for use at the local EM level, there is a need for a framework that utilizes the tightknit relationships of community stakeholders to spread uniform critical disaster-related information via social media before, during, and after a disaster. A key priority of the conceptual Communication Hub Framework is to positively leverage community partnerships with local stakeholders to increase the efficiency of disaster-related communication with local community members. The proposed Communication Hub Framework uniquely utilizes the strong relationships between local emergency management agencies and their community stakeholders to foster positive collaboration and amplify the reach of critical social media messaging during a disaster.

## 2. Background Information

According to the Federal Emergency Management Agency (FEMA), an emergency includes “any incident, whether natural, technological, or human-caused, that necessitates responsive action to protect life or property” [7]. The burgeoning discipline of EM focuses on “the coordination and integration of all activities” needed to prepare for, respond to, recovery from, and mitigate against various types of disasters and emergencies [8]. Compared to EM at the state and federal levels, local EM often involves a closer relationship and exchange of information between the local office and the surrounding community [6]. Local stakeholder organizations are typically social, economic, and political organizations within the community [9]. These organizations often influence community members and can provide EM professionals with insight into the needs of a specific section of the community [10]. The tight relationship between local EM offices, their stakeholder organizations, and the surrounding community influences the effectiveness of communication. Additional factors that affect a local EM agency’s communication needs and abilities include the availability of communication resources, the size of the surrounding community, and the “organizational arrangement” of local EM agencies [5,11]. Due to the variability in local EM agencies' size and available communication resources across the U.S., there is a need for a flexible and scalable framework for information dispersal.

Many events, such as 9/11 and Hurricane Katrina, have shaped the field of EM in the United States [12]. In the years preceding the attacks on 11 September 2001, the National Response Framework (NRF) and the National Incident Management System (NIMS) were enacted. A concept essential to the NRF is disaster response both starts and ends locally [7]. The document places an emphasis on the role local emergency managers and local emergency management departments have in the disaster management process. In addition to the NRF, the NIMS is a comprehensive approach to managing emergency which can be used for any size disaster at all levels of government [13]. The NIMS stresses the importance of engaging with stakeholders from the “whole community” to support inclusive participation the disaster management process that represents the diversity of the community [13]. One of the essential elements of NIMS is the Incident Command System (ICS). The ICS is a framework for communication and coordination during response to an ongoing disaster led by the Incident Commander. Within the ICS, there are five varying sections: “command, operations, planning, logistics, and administration/finance” [14]. A PIO is included as one of the command officers supporting the Incident Commander during disaster response and recovery. PIOs are responsible for communicating incident-related information to the media, the public, and coordinating agencies/organizations [15]. The position's duties can be handled by one individual or supported by several assistants depending on the resources available and the size of the incident [16]. At the local level, the PIO might be a volunteer, an emergency manager, or a communication specialist from an adjacent department such as a law enforcement agency or the local government office. The rise of social media in recent years has required the PIO’s role across all levels of EM to adapt [15]. Due to the rising popularity of social media platforms’, crisis communications have shifted away from being a one-way dialogue between PIOs and the public to a bidirectional conversation between the two entities [17].

Throughout the EM cycle, local emergency managers alert and communicate with their communities in various ways, such as using electronic emergency alert systems, outdoor sirens, the media, and social media [6]. According to the Pew Research Center [18], “seven-in-ten Americans use social media” as a form of contacting individuals, receiving news, and sharing information with others. Some of the most popular social media platforms in the United States (U.S.) include Facebook, Instagram, Snapchat, Twitter, WhatsApp, and YouTube [14]. The widespread usage of social media among Americans has given rise to the increasingly popular utilization of social media in a disaster both as an information source and a channel for crisis communications [14,19,20,21,22].

During disasters, individuals tend to seek information from familiar sources such as family members, friends, and local level news outlets [23]. When familiar channels do not provide sufficient information, individuals seek information from additional official sources to mitigate uncertainty about the ongoing crisis [24]. Individuals seeking disaster-related information from social media are interested in receiving essential facts to support their informed decision-making. The introduction of social media as a crisis communication tool has led the public to expect immediate access to disaster-related information via social media, which comes with both benefits and challenges [25].

### 2.1. Uses and Benefits of Social Media in Emergency Management

In the field of EM, social media offers many uses and benefits such as quick information dispersal, a platform for gathering information from the public, and a method for fostering situational awareness [13]. A study focusing on social media usage by county-level EM agencies found that county EM agencies’ top uses of social media were the following: provide specific information to the public, risk communication (public alerting or reassurance), public relations, counter rumors/misinformation, increase situational awareness, and sharing information with other organizations [26]. Traditionally, PIOs provide the media with disaster-related information, and the media relays the message to the public [15]. Incorporating social media into a local EM department’s communication plan allows emergency managers and PIOs to directly engage in quick information exchange with the public, thus bypassing the traditional unidirectional pathway of information from the media to the public [17]. This adjustment improves the efficiency of information dispersal and prevents potential misrepresentation of information due to the information be posted directly from the source.

In 2013, the Boston Marathon incident forever changed social media’s utilization in disaster response by signifying the transition to using the platforms as crisis communication tools [4]. The introduction of using social media as a communication tool following the incident granted the public the ability to both receive and provide information through various social media platforms. Following the Boston attacks, almost half of Americans (49%) received incident-related information online or by using a mobile device, and over a quarter of Americans (26%) used social media to keep up with the news surrounding the incident [27]. Social media accounts from credible organizations the public trusted (e.g., the city of Boston officials, Massachusetts’ Governor, the Federal Bureau of Investigation (FBI), and law enforcement agencies) provided the public with frequent, direct updates following the incident [28]. The usage of social media as a method of community interaction and information dispersal aided in the overall EM response and recovery efforts to the hallmark incident.

Social media also provides community members the ability to supply information from the incident scene by posting photos, videos, and personal accounts of the incident on social media sites. During the response to the Boston Marathon bombing, social media served as a platform for survivors of the incident to post photos and videos, which were used to aid the FBI’s and the Boston Police Department’s investigation [28,29]. Following the successful use of social media in various aspects of the response to the Boston Marathon Bombing, the increased use of social media in the field raised a need for methods to analyze social media and crowdsourced data. EM officials can use various social media analysis websites, such as Hootsuite, Trendsmap, Google Analytics, TweetArchivist, and Ushahidi Platform, to analyze social media trends [30,31,32]. Crowdsourced information from social media can foster EM officials’ situational awareness of an ongoing disaster [13].

Outside of a disaster, EM agencies, especially at the local level, can utilize social media as a form of community engagement to foster trust and credibility with the public [15]. Establishing credibility with the EM agency’s account before a disaster is essential to the public acknowledging and using the agency’s social media accounts as an information source during a crisis. In response to the California droughts in 2014, the California Drought Task Force, formed from multiple participating agencies, utilized multiple social media platforms such as Facebook, Youtube, and Twitter to inform the public on drought management, amplify the reach of their messaging, and participate in two-way communications with the public [33,34]. The Task Force utilized Facebook as a communication tool to deliver one-way communication messages and as a two-way form of communication by providing the public with the ability to interact with the Task Force via Facebook’s comment capabilities [33]. In addition to the utilization of Facebook and YouTube, the Task Force also used Twitter to quickly disperse information about current drought risks and how the Task Force was managing those risks. The retweeting feature of Twitter helped various agency members of the Task Forces encourage the reconnection of “other citizens with drought risk management information” through retweeting capabilities [33]. The California Drought Task Force’s diverse use of social media platforms demonstrates the advantages of using various social media platforms to connect and disseminate information with a large reaching audience. Additionally, the Task Force’s successes provide an efficient example of stakeholders uniting and collaborating to share uniformed preparedness and disaster-related information to local community members via social media.

### 2.2. Challenges Associated with the Use of Social Media in Emergency Management

Although the use of social media as a communication tool for EM agencies is increasing, social media was not “specifically designed to support emergency response” and crisis communication [5]; thus, there are several potential challenges to consider prior to using social media as a tool for information dispersal during a disaster. Challenges associated with the use of social media in EM include messages containing critical information getting lost in the influx of social media messages, the spread of false information, conflicting messaging from stakeholders, and the communication method’s reliance on cell service and internet access.

With thousands of tweets occurring each second, vital disaster-related messages can get lost in the influx of social media usage. This issue can be mitigated by including hashtags containing keywords related to the incident in social media messages from EM agencies [30]. During the response to Hurricane Isaac in 2012, “governmental agencies, non-governmental agencies, the public, and the news media” used the hashtag Isaac (#Isaac) when sharing incident related messages on social media [31]. By adding hashtags to their messaging, responding agencies were able to help clarify the intent of the messaging and increase the message’s visibility. Additionally, hashtags can serve as a way to unify the community after a disaster. Following the Boston Marathon incident, the hashtag BostonStrong (#BostonStrong) was utilized across social media platforms as a way to unify the community and raise awareness of recovery initiatives such as the One Boston Fund [35]. Within the proposed Communication Hub framework, hashtags can be implemented into the key messages posted by the Hub Coordinator and reposted by participating stakeholder organizations to increase visibility of the message and reinforce the intent of the message.

An increase in social media usage during a disaster combined with the public’s expectation to receive information quickly and the inherent nature of rumor spreading on social media can foster the spread of false disaster-related information. Misleading information on social media platforms requires EM agencies to address the false statements in a timely manner [30]. To provide timely messaging and identify spreading rumors, PIOs and EM officials need to monitor the public’s social media postings constantly. This requires both staff and resources to execute, but social media analysis websites can be utilized to help quicken the process. For federally declared disasters, EM agencies can utilize FEMA’s rumor control webpage to identify disaster-related rumors that might be spreading in their community [36,37]. The proposed Communication Hub framework aids local emergency management agencies with rumor control by involving community stakeholder organizations in the process of identifying the spread of disaster-related rumors on social media. By implementing the conceptual framework, participating stakeholders will have a single contact, the hub coordinator, to report any findings of disaster-related rumors. Providing a single contact to report findings of misinformation and rumors will help streamline the process and notify the hub coordinator that there is a need for messaging to address and correct the rumors misinformation.

Messaging from stakeholders that conflicts with the disaster-related messaging provided by EM agencies can further the spread of false information and confuse the public. Leading up to Hurricane Harvey’s landfall in 2017, the officials representing the City of Houston and the Mayor of Houston were consistently stating there was no evacuation order for the city on both news media and social media platforms [38]. The Governor of Texas initially supported local Houston officials’ decision not to evacuate the city on both news media and social media platforms. During a press conference less than two days before the hurricane-impacted Houston, the Governor changed his public opinion and used Twitter as a platform to encourage Houston residents to consider evacuating the area. This directly contradicted the Twitter messages streaming from Houston officials and the Mayor’s Twitter accounts advising their residents to continue to shelter in place [38,39]. The conflicting evacuation messages on social media left Houston residents puzzled on whether to heed the guidance of local or state officials [38]. These conflicting social media messages highlight the need for uniform social media crisis communications with consistent messaging from both local and state officials. To achieve this, local and state officials and stakeholders must establish communication frameworks prior to a disaster.

An additional issue is a reliance on either cellphone service or internet access to use social media as a communication tool. Vulnerable populations in affected communities, such as older adults and individuals with low socioeconomic status, might lack access to smartphones, portable digital devices, or internet connection needed to receive disaster-related social media messaging [40,41]. Relying solely on social media as a communication tool could exclude certain populations from receiving critical disaster related-information during certain phases of a disaster [41]. Additionally, a disaster could impact the critical infrastructure EM agencies rely on to disseminate information via social media. In the event of a disaster impacting cellphone towers and powerlines, social media may not be an available means of communication to disseminate information to the affected community. Due to the risk of a disaster impacting the critical infrastructure needed for social media messaging and the varying access to social media for certain populations, social media should be used in combination with other communication methods to ensure communication with the public can occur despite a lack in access to social media platforms. The proposed conceptual Communication Hub Framework can be implemented in local EM agencies to simultaneously involve stakeholders in the communication process, while increasing the reach of EM agencies’ disaster-related messaging to community members. Because of the Framework’s reliance on the powerlines and cellphone towers to relay disaster-related messages to the community, it is crucial that the framework is used in conjunction with primary communication methods such as radios and emergency alert systems.

## 3. Methodology

As the use of social media as a crisis communication tool has increased, frameworks and guidelines have been developed to guide the use of social media in crisis and disaster-related communications. The Social Media Emergency Management (SMEM) Guidance Tool, the Crisis and Emergency Risk Communications (CERC) framework, and a conceptual bidirectional framework were analyzed and used as inspiration for the proposed Communication Hub framework.

### 3.1. Social Media Emergency Management (SMEM) Guidance Tool

The Department of Homeland Security’s (DHS) Science and Technology Directorate (S&T) collaborated with FEMA and several public safety and EM professionals to jointly produce the SMEM Guidance Tool [42]. In August of 2020, the SMEM Guidance Tool was publicly released as an online, beta web application intended to provide “first responders, emergency managers, and public information officers” guidance on how to improve their organization’s planning and actual use of social media in emergency operations [42]. At all levels of government, EM officials can access the online tool free of charge [43]. The SMEM Guidance Tool was created to address the gap between the public’s expectation for government agencies to use social media platforms and governmental agencies’ “delivery capabilities” [44].

The SMEM Guidance Tool web application includes the three following customizable resources: “Building a Social Media Business Case”, “Developing a Social Media Plan”, and “Building a Digital Volunteer Program” [45]. The “Building a Social Media Business Case” template aids PIOs, and EM professionals build a business proposal to create and implement a SMEM program [46]. Throughout the template, the users are led to consider their agencies’ established policies; state and local laws regarding social media use; and the resources required to carry out the SMEM plan. Once users obtain approval for social media use by their agency, the “Developing Social Media Plan” template will aid them in creating a plan for managing social media accounts [47]. The template requires users to consider the organization’s social media team’s roles, determine the objectives of using the selected social media platforms, and create account management procedures. Using the “Building a Digital Volunteer Program” template, PIOs and EM professionals can plan to incorporate volunteers into their social media plan to help collect crowdsourced information and monitor social media interactions [48]. The “Building a Digital Volunteer Program” template helps users create a standard operating procedure, establish a memorandum of understanding, and take steps to start a volunteer program [48]. Overall, the SMEM Guidance Tool effectively provides three robust resources that emergency managers and PIOs can customize to fit their agencies’ communication needs.

Although the SMEM tool aims to guide social media implementation into emergency operations, the tool fails to outline the process of dispersing information via social media during an ongoing disaster [42]. The web application lacks details on how to craft social media messages, essential information to include in posts, and how to use partnerships with stakeholders to aid in message amplification. Additionally, the SMEM Guidance Tool fails to address differential outcomes the tool might have at each level of government due to the differing structures and access to resources across EM agencies at various levels of government. An EM agency’s success with using and implementing the plans developed with the SMEM Guidance Tool has the potential to vary, based on the agency’s level of government, organizational structure, and availability of resources. Despite the small gaps in the provided information, the SMEM Guidance Tool supplies emergency managers and PIOs with an easy-to-use platform to create a social media business case, a social media plan, and a digital volunteer program [45]. Due to the functionality of the SMEM tool, aspects of the SMEM tool, such as the varying templates provided by the tool, are utilized in the proposed conceptual framework.

### 3.2. Crisis and Emergency Risk Communications (CERC) Framework

In 2002, the Centers for Disease Control and Prevention (CDC) created the CERC framework and accompanying manual to aid in an organization’s communication of crisis-related information to the public during public health emergencies [49,50]. The CERC framework uniquely merges risk and crisis communication concepts to use both types of communication to form effective messaging during a crisis via the implementation of the framework’s six fundamental principles [51] are in The Principles of the CERC Framework are as follows: “be first, be right, be credible, express empathy, promote action, and show respect” [50]. According to the CERC manual, crisis and emergency risk communication often occurs unexpectedly, and the communication messages are delivered by a spokesperson whom is also impacted by the crisis unfolding [50]. The goal of this type of communication is disperse accurate information in a timely manner that will enable the public to make sound decisions. Throughout the “preparation, initial, maintenance, and recovery” phases of the disaster, the CERC framework calls for communicators to fulfill the framework’s three objectives of community engagement, empower decision-making, and evaluation [50].

In the CERC manual, social media is highlighted as a tool to gather audience feedback, identify information gaps, engage with the community, and identify the spread of misinformation during a crisis [52,53]. Within the CERC framework, social media posts are used to increase the public’s self-efficacy, establish the public’s trust in the responding agency, and collaborate with key stakeholders by reposting credible posts [54]. Although the CDC’s CERC framework aids communicators at all three levels of government, this framework is specifically intended for communicators in the field of public health. Elements of the framework, such as the six principles of CERC, are applicable to effective crisis communications in the field of emergency management; however, there is a need for a framework with qualities of the CERC framework specifically intended for use by EM professionals. The proposed Communication Hub Framework incorporates the CERC framework’s focus on community engagement as well as the framework’s emphasis on the use of social media messages for engaging in both one-way and two-way communication with the community. Despite the CERC framework and the Communication Hub framework having some similarities, they differ widely due to both frameworks being intended for use within specific disciplines, public health and emergency management respectively.

### 3.3. A Conceptual Framework for Developing Solutions That Organise Social Media Information for Emergency Response Teams

**Figure 1:**
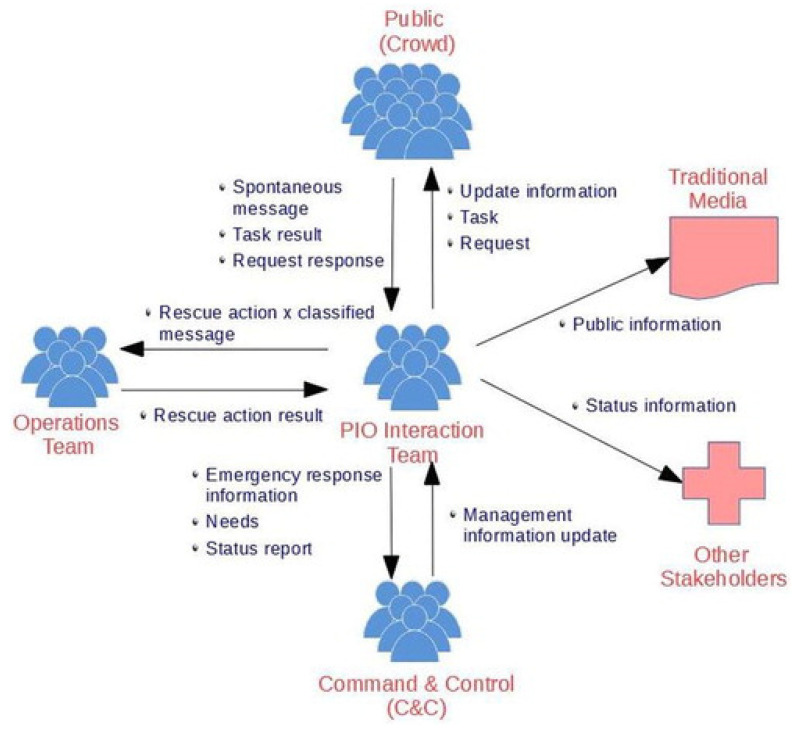
A hub-and-spoke model created and proposed by Freitas et al. in the article titled A Conceptual Framework for Developing Solutions that Organise Social Media Information for Emergency Response Teams [14]. Public Information Officers (PIO). From “A conceptual framework for developing solutions that organise social media information for emergency response teams”, by Freitas, D.P.; Borges, M.R.S.; De Carvalho, P.V.R, 2019, *Behaviour & Information Technology*, Volume *39*, p. 367. Copyright 3 March 2020 by Taylor & Francis. Reprinted with permission of the publisher (Taylor & Francis Ltd., http://www.tandfonline.com).

In the article, “A Conceptual Framework for Developing Solutions that Organise Social Media Information for Emergency Response Teams”, the authors propose a conceptual framework for bidirectional social media interaction during a disaster between the public and key EM officials such as the PIO interaction team, command and control, and the operations team [14]. An essential aspect to the framework is the involvement of a working group, called the PIO interaction team, that focuses on “monitoring, updating, responding and interacting with the public” via social media platforms [14]. Within the paper, Freitas et al. utilized a hub-and-spoke wheel formation to visually demonstrate the flow of information to and from the PIO interaction team (the hub) to the operations team, command and control, and the public (the spokes) as seem in Figure 1 [14]. The authors’ innovative use of the hub-and-spokes wheel formation promotes effective flow of communication from a central team (the PIO Interaction Team). Community stakeholders and traditional media are the only two spokes of the framework that participate solely in unidirectional communication from the PIO interaction team [14]. The unidirectional flow of information to the community stakeholders limits the stakeholders’ involvement in the dispersal of the EM agency’s disaster-related information via social media. Although the framework created by Freitas et al. limits bidirectional communication with stakeholders, the framework’s hub-and-spoke formation has the potential to be adapted to foster bidirectional social media interaction and streamline information dispersal during a disaster.

## 4. Results and Discussion

Following the analyses of previous frameworks and extensive background research of the evolving uses and challenges of using social media as a communication tool during disasters, the authors used the learned lessons and identified gaps to create a conceptual framework: the Communication Hub Framework. Key concepts of previous frameworks, such as the six principles of the CERC framework and the hub-and-spoke model featured in the framework proposed by Freitas et al. [14], are incorporated into the foundation of the Communication Hub Framework. Despite its foundational similarities to previous works, the proposed framework’s uniquely focuses on utilizing stakeholder relationships to enhance unified communication at the local level during a disaster.

### 4.1. A Proposed Conceptual Framework: The Communication Hub Framework

The proposed Communication Hub Framework is specifically intended to aid local level EM professionals, PIOs, and relevant stakeholders in dispersing essential information via social media during a disaster by providing a framework that guides the involvement of community partners in the communication process. Overall, the framework aims to amplify the critical social media messages produced by local EM professionals or PIOs while also ensuring that the public receives consistent, uniform messaging. Stakeholder organizations participating in the Communication Hub will agree to share or repost a local EM agency’s social media messages containing essential information during a disaster, thus ensuring key stakeholders within a community share non-conflicting disaster-related messaging. By stakeholder organizations resharing local EM departments’ original messages, the critical disaster-related messages will be amplified to reach a diverse range of community members. Engaging the community through both the local EM agency’s own social media accounts and stakeholder organizations’ accounts will contribute to the local EM agency’s efforts to maintain a whole community approach to emergency management.

Ideally, the framework would be established during the pre-event stage, activated during the event stage, and evaluated in the post-event stage. Prior to adopting the Communication Hub Framework, local EM departments/agencies should have official social media accounts, such as Facebook and Twitter, and a social media plan in place. Guided templates for creating a social media business proposal and a social media plan can be found using the previously discussed SMEM Guidance Tool. Although the framework was developed with local EM agencies/departments in mind, the framework is scalable and can be adapted for use at higher levels of government.

### 4.2. Main Components of the Communication Hub Framework

**Figure 2:**
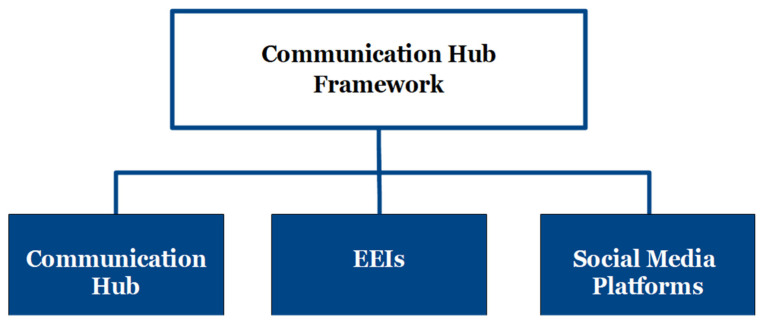
The three main components of the Communication Hub Framework. EEI is defined as Essential Elements of Information.

The main components of the Communication Hub Framework (Figure 2) are the Communication Hub, essential elements of information (EEI), and the specific social media platforms selected by the leading local EM agencies or managers. Five stakeholder organizations and a hub coordinator from the local EM agency comprise the Communication Hub. EEIs, the second main component of the framework, are critical pieces of information needed for informed decision-making [55]. In this case, EEIs are critical information the public needs to know during and after the disaster. The EEI criteria determined by the hub coordinator and agreed upon by the command and coordination team will influence the social media messages created and disseminated by the hub coordinator in a disaster. Lastly, the framework relies on the establishment and use of social media platforms selected by the local EM agency. Upon selecting specific social media platforms, EM professionals should consider their target demographics and the social media platforms to reach their target audiences best.

### 4.3. Main Components of the Communication Hub

**Figure 3:**
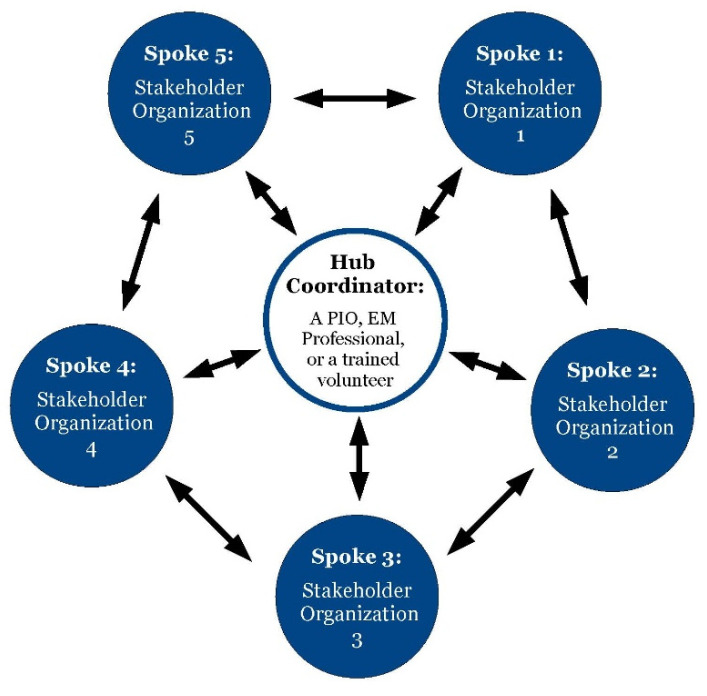
A hub-and-spoke diagram representing the Communication Hub Framework. Emergency Management (EM).

As seen in Figure 3, the hub-and-spoke model proposed by Freitas et al. has been adapted to fit the Communication Hub Framework [14]. Located in the center of the virtual Communication Hub is the hub coordinator, or a single person that represents a larger organization such as local emergency management agency. Examples of hub coordinators include a local PIO, EM professional, or trained volunteer in charge of coordinating communications for the local EM agency or department. Hub Coordinators are responsible for the following: (1) selecting five stakeholder organizations to be spokes of the Communication Hub; (2) determining criteria for EEI to include in social media messages; and (3) communicating and coordinating with the spoke organizations and the public via social media during a disaster.

### 4.4. Selecting “Spoke” Organizations for the Communication Hub

The framework contains five stakeholder organizations as spokes, due to the definition of a manageable span of control in NIMS. According to NIMS, a manageable span of control during an incident is “one supervisor to five subordinates” [13]. Since the framework is scalable, the number of spoke organizations can increase as long as more hub coordinators coordinating uniform messaging are incorporated into the framework. In order to maintain a manageable span of control, an additional hub coordinator would need to be added to the framework for each addition of five more spoke organizations. Both maintaining a manageable span of control and selecting cooperative stakeholder organizations are essential to the framework’s success. When selecting stakeholder organizations to participate in the Communication Hub, hub coordinators should select spoke organizations based on the following characteristics: credibility with their audience, cooperativity, motivation to do the best for the community, and their social media account accessibility. Collectively, the five-spoke organizations should represent the diversity of the community. Examples of spoke organizations include the local school district, civic organizations, law enforcement agencies, religious organizations, and social groups.

When selecting the participating stakeholder organizations as spokes, hub coordinators must ensure that the selected stakeholders are prominent community leaders whom community members trust and a sizeable number of followers. The Communication Hub aims to expand the reach of the local EM agency’s social media messages during a crisis. By selecting stakeholders with unique follower bases, the audience receiving the local EM agency’s critical social media messages during a disaster will expand with the activation of the Communication Hub. In the event of an essential stakeholder lacking prominent social media accounts, the hub coordinator can reach out to the stakeholder organization to gauge their interest in establishing a social media presence and participating in the Communication Hub. Once the spoke organizations are selected, the hub coordinators should establish trustful social media relationships with the spoke organizations and their followers prior to disasters to aid in the efficient use of the Communication Hub during a disaster. Hub coordinators and spoke organizations can foster strong social media relationships by interacting with each other on social media via reposting and commenting on each organization’s posts. This will allow followers of spoke organizations to be introduced to the local EM agency’s social media accounts and vice versa prior to activating the Communication Hub for a disaster event.

### 4.5. Determining Essential Elements of Information (EEI) Criteria

In the pre-event stage, the standardized criteria for EEIs for social media messaging in disasters will be established in the pre-disaster period by the Hub Coordinator, staff within the Hub Coordinator’s local EM agency, and stakeholder organizations participating in the Communication Hub. Established EEI criteria should be included in the agency’s social media messaging template. The social media messaging template should include flexible and plain language that allows the hub coordinator to quickly insert critical information (i.e., time, location, etc.). Additionally, the template should be adaptable for messaging on a variety of social media platforms. Within the template, the established EEI criteria will guide the crafting of the messages distributed to the public via social media.

When determining the EEI criteria, the hub coordinator should consider the information community members must need to make informed decisions such as the impact of the incident, available shelters, evacuation routes, and action steps community members can take [50,56]. Once the hub coordinator establishes the EEI criteria, the criteria should be placed in the local EM agency’s social media communication plan. Social media messages developed and disseminated via the local EM agency’s social media platforms and the spoke organizations platforms should be concise and contain the EEIs community members need to make informed decisions.

### 4.6. Implementing the Communication Hub during a Disaster

**Figure 4:**
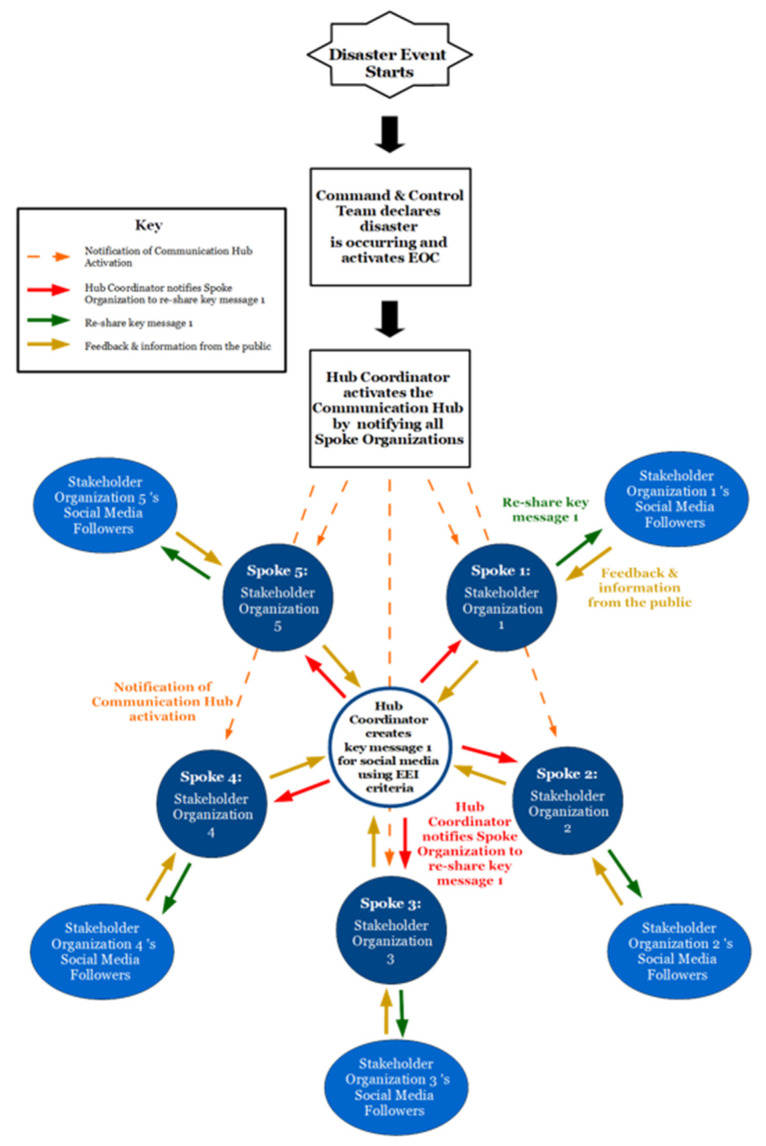
A diagram of how a key social media message would be dispersed from the local EM agency via the Communication Hub during an ongoing disaster event. Emergency Operation Center (EOC).

During a disaster or crisis, the hub coordinator would activate the Communication Hub and notify the participating stakeholder organizations by email, landline, cellphone, or radio (Figure 4). The hub coordinator would use the established EEI criteria to create a concise initial key message to post on the local EM agency’s social media accounts and the spoke organizations’ accounts. As the event progresses, the hub coordinator can create more social media messages as needed. The timing and amount of content shared will be dependent upon identified operational periods and the needs of the event. Once the initial message is crafted, the hub coordinator will release the message on all of the local EM agency’s official social media platforms. The hub coordinator will then notify the spoke organizations of the social media post containing critical disaster-related information via the established methods of communication and post notifications.

As soon as the first message is sent from the local EM agency’s official accounts, the hub coordinator will contact the spoke organizations to have them retweet or reshare the information across all social media platforms. Each spoke organization of the Communication Hub would report any insight or findings back to the hub coordinator. The process will continue as long as the Communication Hub remains active. In the event of spoke organizations reporting findings of misinformation, the hub coordinator will adjust social media messaging to address and correct rumors of misinformation. Any helpful information collected by the stakeholder organizations will be sent to the hub coordinator and passed on to the command team. Following a disaster, the Communication Hub members should hold an after-action review (AAR) meeting to discuss successes, opportunities for improvement, and establish an improvement plan to outline actions needed to improve the Communication Hub’s information dissemination via social media.

### 4.7. Case Study: Hurricane Harvey

In order to further explore the application of the Communication Hub Framework to a real-world situation, the use of social media prior to landfall of Hurricane Harvey was used as a case study. Prior to the devastating landfall of Hurricane Harvey in the city of Houston, Texas, in 2017, messaging on social media from representatives of the City of Houston and the Mayor of Houston had uniformed and constant disaster-related messaging. These local stakeholder organizations consistently stated in both their social media and other media messages that an evacuation order was not in effect for the city in the days leading up to Hurricane Harvey’s landfall [38]. Despite the uniform messages dispersed on social media regarding the advice for Houston residents to shelter in place, the Governor of Texas’ social media messages and his messages to the news media conflicted with the local stakeholder’s guidance. Initially, the Governor of Texas supported local Houston officials’ decision not to evacuate the city on both news media and social media platforms, but he changed his public opinion on the evacuation of the city during a press conference less than two days before the hurricane impacted Houston. At the press conference, the Governor of Texas started encouraging Houston residents to consider evacuating the area. The Governor’s changed opinion was reflected in his social media messaging specifically on Twitter which directly conflicted with the local stakeholder’s messaging on Twitter requesting Houston residents’ shelter in place [38,39]. As a result of the conflicting messages from local and state officials, Houston residents were left confused on whether to follow the guidance of local or state officials [38]. These conflicting social media messages highlight the need for uniform social media crisis communications with consistent messaging from both local and state officials.

The confusion caused by conflicting disaster-related social media messaging could possibly have been avoided by implementing the Communication Hub framework to increase stakeholder involvement, aid in the coordination of uniform messaging among stakeholders, and amplify the uniformed messages across the social media platforms of partnering spoke organizations. In the example of this case, the Hub Coordinator would have been a PIO or emergency manager representing the City of Houston’s local EM agency. Potential spoke organizations would have included the City of Houston, Texas state agencies such as the Texas Emergency Management Agency (TEMA), the Governor of Texas’ administration, and local school districts. The establishment of the Communication Hub Framework and the recruitment of spoke organizations to be a part of the Communication Hub prior to a disaster occurring can help to increase the efficiency and consistency of social media messaging during a disaster.

## 5. Conclusions

Due to the integration of social media into the public’s daily life, social media will continue to grow in popularity as an information source for the public during disasters. The Communication Hub Framework aims to improve local emergency managers and their agency/department’s use of social media to disseminate critical information during a disaster. The framework uniquely incorporates stakeholder organizations as a method of amplifying the reach of critical social media messaging. In addition to the benefits of amplifying the reach of disaster-related messaging, the framework supports the whole community concept and the idea of disaster management starting and ending locally. The proposed conceptual framework fosters positive collaboration between local EM professionals and their stakeholders, while simultaneously working towards disaster risk reduction by establishing a social media communication plan with participating stakeholders prior to the onset of a disaster. Despite the benefits of the framework, there are limitations to the use of the framework as the reliance of power and cell-phone service, dependence on stakeholder engagement and willingness to participate, and the conceptual limitations of the framework. This study is limited by the conceptual constraints of the framework and the lack of research on this framework in practice. The validity of the framework would benefit from future research analyzing the use of the Communication Hub Framework in a real-world exercise or event. However, given that disasters are becoming more frequent by the day it is crucial that local emergency managers have access to critical knowledge, such as the Communication Hub Framework, to provide guidance on how to effectively incorporate stakeholders into the disaster preparedness and response processes. The conceptual Communication Hub Framework effectively provides local emergency managers with a method of utilizing stakeholder partnerships to increase the reach of the critical disaster message on social media as a complementing communication tool to their traditional modes of communication. Although the uses of social media have increased the public’s expectation for quick access to disaster-related information, the Communication Hub Framework can help local EM professionals close the gap between the public’s expectations and their department’s capability to meet the rising expectations.
